# Clinical Factors Associated with Prolonged Hospitalization in Patients Undergoing ERCP: A Tertiary-Care Cohort Study

**DOI:** 10.3390/jcm15187318

**Published:** 2026-09-21

**Authors:** Łukasz Nawacki, Agnieszka Bociek, Ada Bielejewska, Iwona Gorczyca-Głowacka, Stanisław Głuszek

**Affiliations:** 1 Collegium Medicum, Jan Kochanowski University, Aleja IX Wieków Kielc 19, 25-369 Kielce, Poland; 2 Department of Anesthesiology and Intensive Care, Center of Postgraduate Medical Education, Orlowski Hospital, 00-416 Warsaw, Poland; 3 Institute of Genetics and Animal Biotechnology of the Polish Academy of Sciences, 05-552 Jastrzębiec, Poland; 4 Department of Surgical Oncology, Holy Cross Cancer Centre in Kielce, 25-734 Kielce, Poland; 5 Department of Surgery, Hospital of the Ministry of Interior and Administration in Kielce, 25-375 Kielce, Poland

**Keywords:** ERCP, length of stay, prolonged hospitalization, post-ERCP pancreatitis, hospital utilization, clinical factors

## Abstract

**Background/Objectives:** Hospital length of stay (LOS) in patients undergoing endoscopic retrograde cholangiopancreatography (ERCP) may reflect baseline condition and underlying disease as well as procedure-related morbidity. We evaluated factors associated with prolonged hospital stay (PLOS) and the association of post-ERCP pancreatitis (PEP) with hospitalization duration. **Methods:** This retrospective single-center secondary analysis included 806 consecutive patients undergoing ERCP within 48 h of admission in 2019–2021. PLOS was defined as total LOS ≥ 12 days, corresponding to hospitalization above the 75th percentile. Modified Poisson regression estimated adjusted risk ratios (aRRs); LOS was also analyzed using negative binomial and median quantile regression. Sensitivity analyses used LOS > 7 and >14 days, a PLOS/death composite, and a PEP-excluded cohort. **Results:** Among 788 live discharges with complete LOS data, 176 (22.3%) met the PLOS criterion. In the primary Model A, emergency admission (aRR 3.01, 95% CI 2.01–4.51), cholangitis/postoperative indication (aRR 2.71, 95% CI 1.57–4.66), liver disease (aRR 1.85, 95% CI 1.10–3.12), and age (aRR 1.12 per 10 years, 95% CI 1.02–1.22) were associated with PLOS. PEP was associated with PLOS when added as a post-procedural exposure (aRR 2.34, 95% CI 1.67–3.29). Baseline associations were broadly preserved across sensitivity analyses. **Conclusions:** Prolonged hospitalization was associated mainly with acute clinical context, underlying disease, and patient characteristics. PEP was also associated with longer hospitalization, but this result should be interpreted cautiously because the historical PEP adjudication rule was not retained.

## 1. Introduction

Endoscopic retrograde cholangiopancreatography (ERCP) is a central therapeutic procedure in interventional pancreatobiliary medicine. Its contemporary role is predominantly therapeutic, encompassing biliary drainage, stone extraction, treatment of benign and malignant strictures, stent placement, and selected pancreatic duct interventions [1,2,3]. Despite advances in technique and peri-procedural care, ERCP remains associated with clinically relevant adverse events. A recent systematic review and meta-analysis of 380 randomized and large observational studies reported pooled incidences of post-ERCP pancreatitis (PEP), clinically significant bleeding, cholangitis, cholecystitis, and perforation of 4.6%, 1.5%, 2.5%, 0.8%, and 0.5%, respectively, with ERCP-attributable mortality of approximately 0.2% [4]. A contemporary review further emphasized the downstream resource burden, estimating that unplanned hospitalization may occur in up to one in six patients undergoing ERCP, predominantly for evaluation and management of adverse events [5]. These data underline that the burden associated with ERCP extends beyond technical success.

Hospital utilization after ERCP is not fully captured by conventionally defined procedural adverse events. In a secondary analysis of high-risk outpatients, Yachimski et al. reported that 21.9% of patients initially discharged after ERCP required hospitalization within 30 days [6]. In a prospective multicenter cohort, Thiruvengadam et al. found a clinically meaningful increase in patient-reported morbidity in 21.6% of patients seven days after ERCP, whereas only 4.7% fulfilled criteria for PEP; many patients with increased morbidity therefore had no formally diagnosed PEP [7]. At the population level, Radadiya et al. reported a 12.5% all-cause 30-day readmission rate after inpatient ERCP, with comorbidity burden and cirrhosis among the associated factors [8]. Together, these findings support hospital utilization and length of stay as clinically relevant outcomes in their own right.

Prolonged hospitalization in patients undergoing ERCP is likely to reflect the interaction of baseline vulnerability, underlying pancreatobiliary disease, procedural intervention, and post-procedural morbidity. In a nationwide analysis of more than 1.1 million inpatient ERCP admissions, Leslie et al. found substantially longer hospitalization and worse outcomes among patients with chronic kidney disease, congestive heart failure, or liver cirrhosis [9]. Procedure complexity may also contribute: Goubert et al. identified anticoagulant therapy and higher procedural difficulty as independent factors associated with severe ERCP-related complications [10]. In our previous analysis of the same 806-patient cohort, which focused specifically on PEP, PEP occurred in 7.4% of patients and was associated with longer hospitalization [11]. That analysis, however, did not address prolonged hospitalization as the primary outcome within an integrated patient-, disease-, and procedure-related framework.

Accordingly, the determinants of prolonged hospitalization itself remain incompletely characterized, particularly in real-world inpatient cohorts in which baseline clinical context and procedural factors are evaluated simultaneously. The present secondary analysis therefore aimed to identify patient-, disease-, and procedure-related factors associated with prolonged hospitalization among patients undergoing ERCP in a tertiary-care setting. A second objective was to assess the association of PEP with prolonged hospitalization and total LOS after accounting for factors identifiable before or during ERCP. The analyses were designed to estimate explanatory associations rather than to develop or validate a clinical prediction model.

## 2. Materials and Methods

### 2.1. Study Design and Setting

This retrospective, single-center observational study was a secondary analysis of an institutional cohort of consecutive patients undergoing ERCP between 1 January 2019 and 31 December 2021. The source cohort and its PEP-focused analysis have been reported previously [11]. Each database record represented a unique patient. The present study addressed a distinct research question: hospitalization duration was the primary outcome domain, whereas PEP was treated as an additional post-procedural exposure rather than the primary endpoint. The models were developed to estimate adjusted associations and were not intended as clinical prediction models.

The original cohort comprised 806 patients. The study population, selection criteria, procedural management, and source database were unchanged from the previous cohort report [11]. No formal sample-size calculation was performed for the present secondary analysis; all eligible patients available in the source cohort were considered. The previous report included LOS as a secondary clinical outcome but did not evaluate determinants of prolonged hospitalization using the dedicated multivariable framework applied in the present study.

### 2.2. Study Population and Eligibility Criteria

Patients were included if ERCP was performed within 48 h of hospital admission. Patients with ERCP delayed beyond 48 h were excluded from the source cohort to reduce heterogeneity related to pre-procedural hospitalization and progression of the underlying disease. As previously reported, delays could arise from the need to correct coagulation abnormalities, stabilize severe cardiac or respiratory comorbidity, or from temporary organizational and operating-room constraints [11]. ERCP was available during regular working hours from Monday to Friday (08:00–13:00).

Patients undergoing ERCP in the presence of active acute pancreatitis were also excluded. For the primary PLOS analysis, patients who died during the index hospitalization were excluded because death precludes conventional live discharge and can truncate observed LOS. A sensitivity analysis subsequently used a composite outcome of PLOS or in-hospital death.

### 2.3. ERCP Procedure and Periprocedural Management

All ERCP procedures were performed by the same experienced operator under general anesthesia according to the standardized institutional protocol used throughout the study period. Pharmacological prophylaxis consisted of diclofenac 100 mg administered rectally and cefazolin 2 g administered intravenously approximately 30 min before ERCP [11].

Therapeutic interventions recorded in the database included endoscopic sphincterotomy and biliary stent management. Stent status was categorized as no stent, plastic stent, self-expandable metal stent (SEMS), or stent removal during the index ERCP. The source database did not systematically capture cannulation difficulty, number or duration of cannulation attempts, precut access, unintended pancreatic duct cannulation or contrast injection, pancreatic guidewire passages, total procedure duration, or a validated ERCP difficulty score; these variables could not therefore be included in the present analysis.

### 2.4. Data Collection and Variable Definitions

Demographic, clinical, disease-related, procedural, and outcome data were obtained from the institutional ERCP database and available medical documentation. Patient-related variables evaluated in the explanatory models were age, sex, admission mode, previous ERCP, history of acute pancreatitis, previous cholecystectomy, gallbladder stones, liver disease, diabetes mellitus, and obesity. Diabetes was analyzed as a binary comorbidity irrespective of recorded subtype. The database recorded individual comorbidities rather than a validated cumulative index such as the Charlson or Elixhauser Comorbidity Index.

ERCP indications were grouped before model fitting into four clinically interpretable categories: (1) choledocholithiasis, used as the reference category; (2) malignant obstruction, including pancreatic, bile duct, ampullary, gallbladder, and hilar malignant lesions; (3) benign disease/reintervention, including benign papillary or biliary stenosis, planned reintervention or stent removal, chronic pancreatitis, and other rare benign indications; and (4) cholangitis/postoperative complications. Procedure-related variables included sphincterotomy and stent status, with no stent as the reference category.

PEP status was carried forward from the previously curated source cohort and was not re-adjudicated for the present secondary analysis [11]. The archived analysis dataset did not retain a separate field specifying the diagnostic adjudication rule used for each PEP case. PEP was therefore analyzed as the recorded post-procedural clinical diagnosis from the source cohort. Among patients with PEP, severity had been classified according to the revised Atlanta classification [12].

Hospital length of stay (LOS) was defined as total duration of the index hospitalization from admission to discharge, expressed in days. It should therefore not be interpreted as the exact ERCP-to-discharge interval. Because all included procedures were performed within 48 h of admission, LOS reflects the overall inpatient episode in which ERCP was performed.

### 2.5. Study Outcomes

The primary outcome for the present secondary analysis was prolonged hospital stay (PLOS), defined before multivariable model fitting as total LOS ≥ 12 days. This threshold corresponds to hospitalization beyond the 75th percentile of the observed LOS distribution; the third quartile was 11 days. No externally validated LOS cutoff exists for this inpatient ERCP setting, and absolute LOS is strongly influenced by case mix and local care organization. We therefore used the upper quartile of the cohort distribution to identify patients with relatively long hospitalizations, without proposing this as a universal clinical cutoff. To assess threshold dependence, sensitivity analyses additionally evaluated LOS > 7 days and LOS > 14 days.

Secondary analyses treated LOS as its original count outcome and as the conditional median. To examine whether excluding deaths materially influenced inference, a sensitivity outcome combined PLOS (LOS ≥ 12 days) with in-hospital death. Because PEP occurs after ERCP whereas LOS begins at hospital admission, Model A and Model B were interpreted as answering distinct temporal questions rather than as conventional confounder-adjustment steps.

### 2.6. Statistical Analysis

Continuous variables were summarized as means with standard deviations or medians with interquartile ranges (IQRs), as appropriate. Categorical variables were reported as frequencies and percentages. Between-group comparisons used the Mann–Whitney U test for continuous variables and Pearson’s chi-square test or Fisher’s exact test for categorical variables, as appropriate. All tests were two-sided, with *p* < 0.05 considered statistically significant.

The principal multivariable analysis used modified Poisson regression with an HC0 heteroskedasticity-consistent covariance matrix to estimate adjusted risk ratios (aRRs) and 95% confidence intervals (CIs) for PLOS. Covariates were selected on clinical grounds before fitting the multivariable models rather than by automated stepwise selection. Model A was the primary explanatory model and included factors available before or during ERCP: age, sex, emergency versus elective admission, gallbladder stones, previous cholecystectomy, previous ERCP, history of acute pancreatitis, liver disease, diabetes, obesity, grouped ERCP indication, sphincterotomy, and stent status. Model B was a sequential secondary model that additionally incorporated PEP as a post-procedural exposure. Age was analyzed continuously and reported per 10-year increase. Multicollinearity was assessed using variance inflation factors (VIFs).

Because LOS was right-skewed and the Poisson model showed substantial overdispersion, LOS was additionally analyzed using negative binomial regression with an NB2 variance structure and log link. Results were expressed as incidence rate ratios (IRRs) with 95% CIs. Median quantile regression (τ = 0.50) with robust covariance estimation and Hall–Sheather bandwidth selection was used to estimate adjusted differences in median LOS directly in days. The same covariate structure as Model B was used for both analyses. Sensitivity analyses refitted the modified Poisson models using LOS > 7 days and LOS > 14 days. To examine whether the baseline associations depended on the PEP subgroup, Model A was also refitted after excluding all patients with PEP.

Missing values were handled by complete-case analysis. One patient had no originally recorded LOS and was excluded from analyses requiring LOS. One additional patient had an unclassifiable admission mode because neither elective nor emergency status was recorded and was excluded from multivariable models containing admission mode. No missing outcome or covariate values were imputed. The primary descriptive PLOS cohort therefore included 788 patients, whereas the multivariable PLOS and continuous-LOS models included 787 patients. The composite sensitivity analysis included 804 complete cases; the PEP-excluded complete-case sensitivity cohort included 731 patients. No adjustment for multiplicity was applied; secondary and sensitivity analyses were considered supportive.

Model adequacy was summarized using measures appropriate to each model family. For the modified Poisson models, Akaike information criterion (AIC), McFadden pseudo-R^2^, deviance explained, and root mean square error (RMSE) were reported as measures of in-sample fit. For the negative binomial model, AIC, McFadden pseudo-R^2^, RMSE, Pearson χ^2^/df, and the NB2 dispersion parameter (alpha) were examined. For median quantile regression, the Koenker–Machado pseudo-R^2^ was reported. These measures were used to describe model fit in the analyzed sample; no internal or external predictive validation was performed, and the models were not intended for bedside prediction.

All final statistical analyses reported in this manuscript were reproduced from the original source workbook using Python 3.13.5 (Python Software Foundation, Wilmington, DE, USA), with statsmodels 0.14.6, SciPy 1.17.0, pandas 2.2.3, and NumPy 2.3.5.

### 2.7. Use of Generative Artificial Intelligence

During the preparation of this manuscript, the authors used OpenAI GPT-5.6 Sol (San Francisco, CA, USA) to assist with manuscript structuring, Polish-to-English translation, and English-language editing. The tool was not used to generate study data or to make clinical or scientific decisions. All AI-assisted output was critically reviewed, verified, and edited by the authors, who take full responsibility for the accuracy, integrity, and final content of the manuscript.

### 2.8. Ethical Considerations

The present study constitutes a secondary analysis of the same retrospectively collected institutional cohort that received ethical approval for the previously published PEP investigation [11]. No additional study-specific intervention or prospective patient contact was undertaken.

The study was conducted in accordance with the Declaration of Helsinki and approved by the Bioethical Committee of The Jan Kochanowski University in Kielce (protocol code 27/2023, dated 19 May 2023). Because of the retrospective nature of the study, the requirement for individual informed consent was waived. Results are reported in aggregate form.

## 3. Results

### 3.1. Study Population and Length-of-Stay Distribution

**Figure 1:**
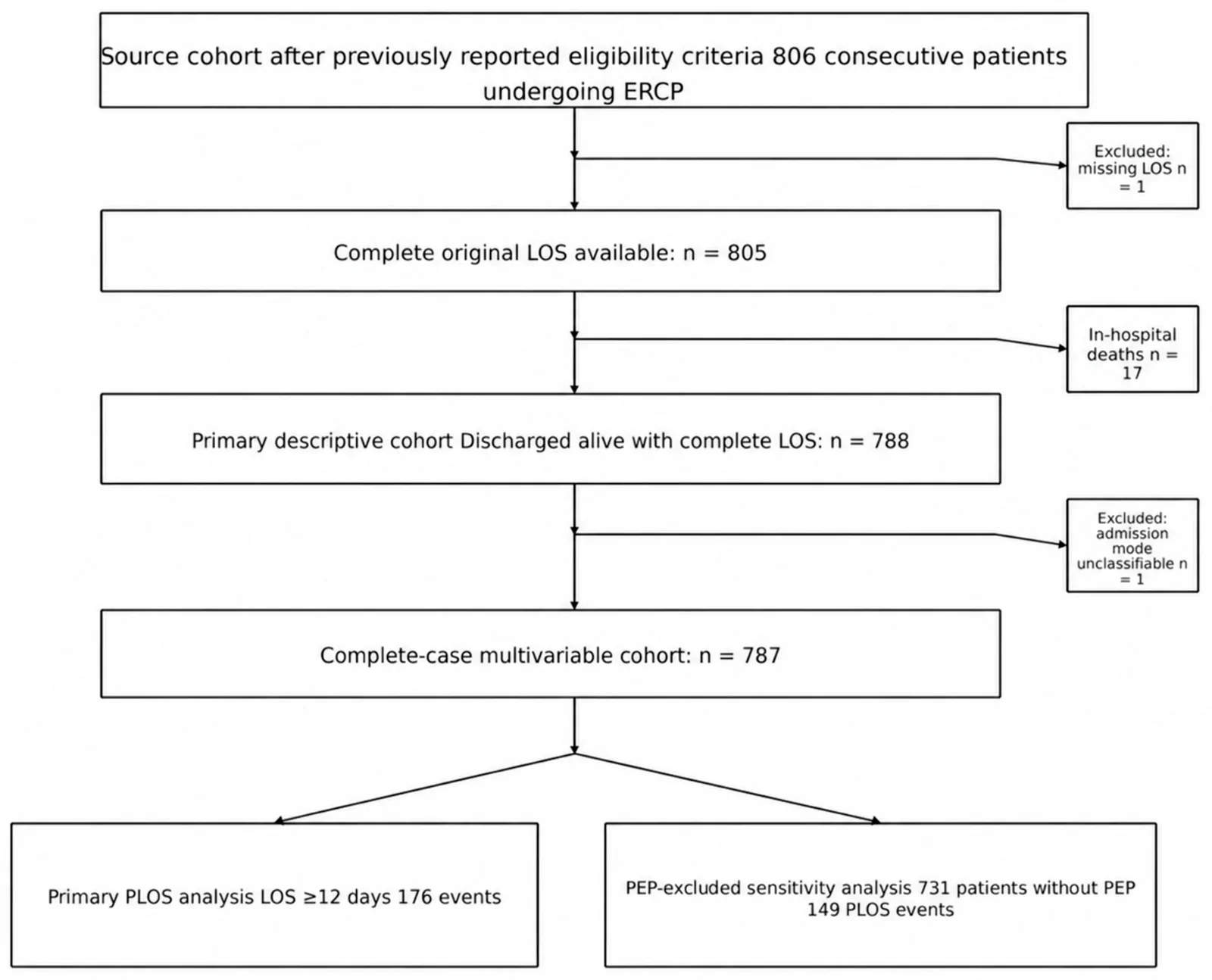
Study flow diagram. The source cohort comprised 806 patients after application of the previously reported eligibility criteria [11]. The archived secondary-analysis dataset did not retain the number of procedures screened before construction of this source cohort; the diagram therefore begins with the established 806-patient cohort and shows exclusions specific to the present analysis. The primary descriptive cohort included 788 patients discharged alive with complete LOS data, and complete-case multivariable models included 787 patients. ERCP, endoscopic retrograde cholangiopancreatography; LOS, length of stay; PEP, post-ERCP pancreatitis; PLOS, prolonged hospital stay.

The source cohort comprised 806 patients. One patient had no originally recorded LOS, leaving 805 patients with complete LOS data. Seventeen patients (2.1%) died during the index hospitalization, leaving 788 patients discharged alive with complete LOS data for the primary descriptive analysis. Median LOS among the 805 patients with available LOS was 7 days (IQR, 4–11), mean LOS was 8.63 days, the observed range was 1–51 days, and the 90th percentile was 16 days. PLOS (LOS ≥ 12 days) occurred in 176/788 patients (22.3%). The study flow is summarized in Figure 1.

### 3.2. Unadjusted Associations with Prolonged Hospital Stay

Patients meeting the PLOS criterion were older and were substantially more likely to have been admitted emergently, to have liver disease, to undergo sphincterotomy, and to have PEP (Table 1). Among patients with a known admission mode, PLOS occurred in 31.2% of emergency admissions compared with 8.0% of elective admissions (*p* < 0.001). It occurred in 48.2% of patients with PEP versus 20.4% of those without PEP (*p* < 0.001). PLOS also varied by ERCP indication (global *p* = 0.001) and stent status (global *p* < 0.001). Previous ERCP, previous cholecystectomy, and a history of acute pancreatitis were associated with lower PLOS rates in unadjusted comparisons.

### 3.3. Multivariable Analysis of Prolonged Hospital Stay

The complete-case multivariable cohort included 787 patients. In the primary explanatory Model A, emergency admission was the strongest factor associated with PLOS (aRR 3.01, 95% CI 2.01–4.51; *p* < 0.001). Cholangitis/postoperative indication (aRR 2.71, 95% CI 1.57–4.66; *p* < 0.001), liver disease (aRR 1.85, 95% CI 1.10–3.12; *p* = 0.021), and increasing age (aRR per 10 years 1.12, 95% CI 1.02–1.22; *p* = 0.013) were also independently associated with PLOS (Table 2).

**Figure 2:**
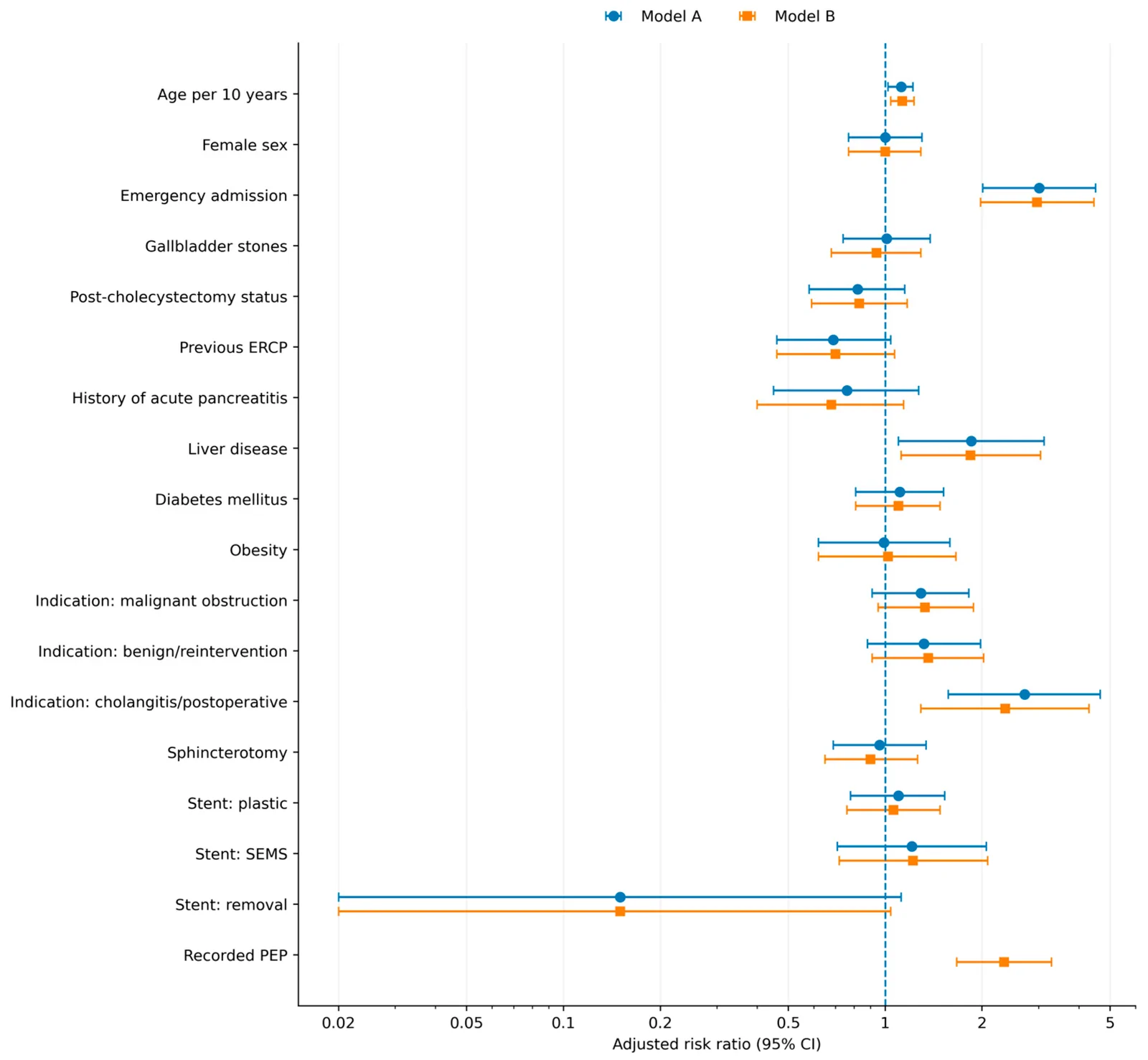
Adjusted risk ratios and 95% confidence intervals for PLOS from Model A and Model B (complete-case *n* = 787). Model A included factors available before or during ERCP; Model B additionally incorporated PEP as a post-procedural exposure. The dashed reference line denotes an adjusted risk ratio of 1.0. The *x*-axis is displayed on a logarithmic scale.

When PEP was additionally incorporated in the sequential Model B as a post-procedural exposure, the baseline associations remained similar: emergency admission (aRR 2.96, 95% CI 1.98–4.45; *p* < 0.001), cholangitis/postoperative indication (aRR 2.36, 95% CI 1.29–4.30; *p* = 0.005), liver disease (aRR 1.84, 95% CI 1.12–3.04; *p* = 0.017), and age (aRR per 10 years 1.13, 95% CI 1.04–1.23; *p* = 0.003) remained associated with PLOS. PEP was itself associated with PLOS (aRR 2.34, 95% CI 1.67–3.29; *p* < 0.001). No problematic multicollinearity was identified (maximum VIF 1.69). Model A and Model B estimates are compared graphically in Figure 2.

### 3.4. Adjusted Analyses of Hospital Length of Stay

The Poisson model for LOS showed substantial overdispersion (Pearson dispersion 3.28); negative binomial regression was therefore used. In the fully adjusted model, emergency admission was associated with a 74% higher expected LOS (IRR 1.74, 95% CI 1.59–1.90; *p* < 0.001), and PEP with a 62% higher expected LOS (IRR 1.62, 95% CI 1.40–1.87; *p* < 0.001). Longer expected LOS was also associated with cholangitis/postoperative indication (IRR 1.45, 95% CI 1.20–1.76), liver disease (IRR 1.35, 95% CI 1.09–1.68), malignant obstruction (IRR 1.16, 95% CI 1.04–1.30), and increasing age (IRR per 10 years 1.05, 95% CI 1.02–1.08). Stent removal was associated with shorter expected LOS (IRR 0.71, 95% CI 0.59–0.86) (Table 3). These estimates describe associations with total hospitalization duration and should not be interpreted as causal effects.

Median quantile regression yielded a similar clinical pattern. Emergency admission was associated with an adjusted median LOS difference of +3.95 days (95% CI +3.34 to +4.57; *p* <0.001), and PEP with +3.46 days (95% CI +2.36 to +4.56; *p* <0.001). Cholangitis/postoperative indication (+2.47 days), malignant obstruction (+1.06 days), diabetes (+0.85 days), and age (+0.35 days per 10 years) were associated with higher adjusted median LOS, whereas previous ERCP was associated with a modestly shorter median LOS (−0.81 days).

### 3.5. Sensitivity Analyses

To address the possibility that excluding in-hospital deaths could bias the PLOS analysis, a composite outcome of LOS ≥12 days or in-hospital death was evaluated. The complete-case sensitivity cohort included 804 patients, with 193 composite events. The principal associations were preserved: emergency admission (aRR 2.56, 95% CI 1.79–3.65), PEP (aRR 2.34, 95% CI 1.71–3.20), age (aRR per 10 years 1.13, 95% CI 1.05–1.22), liver disease (aRR 1.84, 95% CI 1.17–2.89), and cholangitis/postoperative indication (aRR 2.30, 95% CI 1.32–3.99) remained associated with the composite endpoint. Malignant obstruction was also associated with the composite outcome (aRR 1.59, 95% CI 1.14–2.21).

Threshold sensitivity analyses showed that the principal findings were not dependent on the 12-day definition (Table 4). Among the 788 live discharges, 364 (46.2%) had LOS > 7 days and 105 (13.3%) had LOS > 14 days. In complete-case Model B analyses (*n* = 787), emergency admission remained associated with both LOS > 7 days (aRR 2.41, 95% CI 1.91–3.04) and LOS > 14 days (aRR 2.57, 95% CI 1.55–4.27). Cholangitis/postoperative indication and PEP were also associated across both alternative thresholds. Liver disease was not associated with the >7-day threshold but was associated with LOS > 14 days (aRR 2.10, 95% CI 1.02–4.32).

To assess whether the principal baseline findings were driven by the PEP subgroup, Model A was refitted after excluding all patients with PEP. Among 731 complete-case patients without PEP, 149 met the PLOS criterion. Emergency admission (aRR 3.22, 95% CI 2.05–5.07), increasing age (aRR per 10 years 1.20, 95% CI 1.10–1.32), malignant obstruction (aRR 1.50, 95% CI 1.02–2.22), and cholangitis/postoperative indication (aRR 3.83, 95% CI 2.20–6.69) remained associated with PLOS. The liver-disease estimate remained above unity but was attenuated and no longer statistically significant (aRR 1.53, 95% CI 0.85–2.75). The stent-removal coefficient was not estimable in this sensitivity analysis because no PLOS events occurred in that subgroup.

### 3.6. Model Adequacy Diagnostics

Descriptive model-adequacy measures are summarized in Table 5. For PLOS, Model B had a lower AIC than Model A (821.3 vs. 831.7), with modestly higher McFadden pseudo-R^2^ (0.109 vs. 0.095) and deviance explained (18.2% vs. 15.8%); RMSE was 0.389 and 0.393, respectively. For the NB2 LOS model, Pearson χ^2^/df was 1.24 and the estimated dispersion parameter alpha was 0.189, supporting adequate handling of overdispersion. The median quantile regression pseudo-R^2^ was 0.190. These measures describe model fit in the analyzed sample and do not constitute internal or external validation.

## 4. Discussion

In this tertiary-care cohort, 22.3% of live-discharged patients with complete LOS data met the study-defined PLOS criterion. This proportion reflects the distribution in this cohort and should not be interpreted as a general prevalence estimate of prolonged hospitalization among patients undergoing ERCP. The main finding was that longer hospitalization was more strongly associated with clinical context than with the limited procedural variables available in the dataset. Emergency admission showed the strongest baseline association, while cholangitis/postoperative indications, liver disease, and increasing age were associated with PLOS in the primary Model A. Adding PEP in Model B had little effect on these estimates. Similar patterns were seen in continuous LOS analyses, the composite PLOS/death endpoint, the >7-day and >14-day thresholds, and the analysis excluding PEP. Taken together, these results suggest that the main findings are not dependent on a single LOS cutoff or on the PEP subgroup.

The magnitude of the association with emergency admission highlights the importance of acuity and case mix. Emergency admission was associated with approximately a threefold adjusted risk of PLOS, a 74% higher expected LOS, and nearly four additional median hospital days. Emergency status is likely to capture a broader burden of acute illness than can be represented by individual diagnoses in a retrospective database. This interpretation is consistent with Leslie et al., who analyzed more than 1.1 million inpatient ERCP admissions and reported mean LOS of 8.5 days in patients with chronic kidney disease, congestive heart failure, or liver cirrhosis compared with 5.3 days in lower-risk patients [9]. Our cohort had a mean LOS of 8.63 days, which is closer to the high-risk group in that national analysis and likely reflects the tertiary inpatient case mix, the high proportion of emergency admissions, and the use of total admission-to-discharge LOS rather than post-procedure LOS. It also aligns with the nationwide analysis by Radadiya et al., in which comorbidity burden and cirrhosis were associated with 30-day readmission after inpatient ERCP [8]. In our cohort, liver disease was uncommon but associated with the primary PLOS outcome and longer expected LOS, whereas cholangitis/postoperative indications showed a particularly strong association. These patients frequently require treatment of infection, physiologic derangement, postoperative complications, or additional interventions in parallel with biliary decompression; ERCP is therefore only one component of a broader acute-care episode.

Malignant biliary obstruction showed a more nuanced relationship with hospitalization. It did not reach statistical significance for the binary ≥12-day PLOS outcome after full adjustment, yet it was associated with longer LOS in both negative binomial and median regression and with the composite PLOS/death endpoint. It also became associated with PLOS when patients with PEP were excluded. This illustrates the information lost when a continuous outcome is dichotomized at a single threshold: a systematic shift toward longer hospitalization may be clinically relevant without moving a sufficiently large proportion of patients beyond an empirical cutoff. The observation is also compatible with prospective data from Thiruvengadam et al., in which pancreatic adenocarcinoma was strongly associated with clinically significant post-ERCP symptom burden [7]. Real-world data from 596 ERCP-based treatments for malignant biliary obstruction further illustrate the complexity of this population, with reintervention required in 27% of patients and complications recorded in 15% within 30 days [13]. Malignant obstruction should therefore be viewed as a marker of a more complex inpatient course rather than as a binary predictor of crossing a universal PLOS threshold.

PEP was also associated with hospital utilization. In Model B, PEP was associated with a 2.34-fold risk of PLOS, a 62% higher expected LOS, and approximately 3.46 additional median hospital days. Model B, however, addresses a different temporal question from Model A because PEP occurs after ERCP whereas LOS is measured from admission. In addition, the archived secondary-analysis dataset did not retain the diagnostic adjudication rule used for individual PEP cases, and widely used PEP definitions incorporate new or prolonged hospitalization [7,14]. The observed PEP–LOS association may therefore be partly circular and potentially bidirectional: PEP may prolong hospitalization, while a longer or more complicated hospital course may also have made a PEP diagnosis more likely to be assigned or retained in the source dataset. Accordingly, this estimate should be treated as an association with the PEP diagnosis recorded in the source cohort rather than as a causal effect. When all PEP cases were excluded, emergency admission, increasing age, malignant obstruction, and cholangitis/postoperative indication remained associated with PLOS, showing that the principal baseline findings were not driven by the PEP subgroup. Contemporary PEP guidelines continue to emphasize prevention and management because PEP remains a major adverse event after ERCP [15], but patient-reported data also show that formal PEP diagnoses capture only part of post-ERCP morbidity. The discordance between PEP rates and patient-reported morbidity described by Thiruvengadam et al. suggests that LOS may capture dimensions of illness and post-ERCP burden that are not formally diagnosed as PEP [7].

The available procedural variables contributed less strongly after adjustment, but this should not be interpreted as evidence that technical complexity is unimportant. Sphincterotomy and biliary stent placement were associated with PLOS in unadjusted comparisons, whereas neither sphincterotomy nor placement of a plastic or self-expandable metal stent remained independently associated with the primary outcome. These recorded variables are crude proxies for procedural complexity and are susceptible to confounding by indication. The database did not systematically capture cannulation difficulty, number or duration of cannulation attempts, precut access, unintended pancreatic duct cannulation or contrast injection, pancreatic guidewire passages, total procedure duration, or a validated difficulty score. Yachimski et al. identified pancreatic duct cannulation and other technical features as relevant to subsequent hospitalization [6], and Goubert et al. found higher ERCP difficulty to be strongly associated with severe complications [10]. Therefore, a study with comprehensive procedural data would be required to estimate the independent contribution of technical complexity to hospitalization duration. Conversely, the association of stent removal with shorter expected LOS should not be interpreted as a protective biological effect, because planned stent removal commonly represents a scheduled reintervention in clinically stable patients.

From a clinical perspective, the findings suggest that the probability of a prolonged inpatient episode is partly associated with information available before or during ERCP. Patients admitted emergently, particularly older individuals and those with liver disease, cholangitis, postoperative biliary complications, or malignant obstruction, may require more extensive inpatient care irrespective of whether PEP subsequently occurs. These observations may inform discharge planning, resource allocation, and counseling about the expected course of hospitalization. The present models were explicitly explanatory rather than predictive. Although descriptive model-adequacy measures were reported, no internal or external validation was performed, and the models should not be used as a bedside risk score. Development of a clinical prediction tool would require prospective multicenter data incorporating validated comorbidity indices, frailty, physiologic severity, and detailed ERCP complexity.

Several strengths merit emphasis. The cohort comprised more than 800 consecutive patients managed in a highly standardized procedural environment. ERCP was performed within 48 h of admission, all procedures were undertaken by a single experienced operator under general anesthesia, and the same pharmacologic prophylaxis was used during the study period. The statistical strategy also avoided reliance on a single dichotomized endpoint: modified Poisson regression quantified relative risk of PLOS, while negative binomial and median quantile regression evaluated LOS without dichotomization. Sensitivity analyses using >7-day and >14-day thresholds, a composite PLOS/death outcome, and exclusion of all PEP cases further assessed robustness. The consistency of the principal baseline associations across these complementary analyses supports the internal robustness of the findings.

The study also has important limitations. First, its retrospective single-center design precludes causal inference and limits generalizability. Second, the restriction to ERCP performed within 48 h of admission is especially relevant to an LOS study. Patients whose procedures were delayed because of coagulopathy, cardiopulmonary instability, or organizational constraints were not represented, and some may have had particularly complex or prolonged hospitalizations. Third, the database lacked a validated measure of overall comorbidity or frailty, including Charlson or Elixhauser scores, ASA physical status, and systematically defined chronic kidney disease, congestive heart failure, or anticoagulant exposure. Because the available conditions do not constitute a validated cumulative index, an ad hoc comorbidity score was not constructed; residual confounding by overall comorbidity burden cannot be excluded. Fourth, the available procedure variables were limited and should not be taken to show that technical complexity is unimportant. Although the single-operator setting reduced procedural heterogeneity, it may also limit generalizability to centers with multiple endoscopists and broader variation in technical practice. Fifth, the database did not systematically capture all post-ERCP adverse events as dedicated variables, such as bleeding, perforation, or post-procedural cholangitis. Sixth, uncertainty regarding historical PEP adjudication creates potential circularity and bidirectionality between PEP status and LOS; the PEP findings are therefore supportive and non-causal. Seventh, LOS was measured from admission to discharge rather than from ERCP to discharge, so part of LOS reflects pre-procedural care and treatment of the underlying disease. Eighth, the ≥12-day PLOS definition was derived from this cohort and is not a clinically established or externally validated cutoff. The alternative-threshold and continuous-LOS analyses reduce, but do not eliminate, this limitation. Ninth, exclusion of in-hospital deaths from the primary PLOS analysis may underestimate hospitalization burden among the highest-risk patients because death can truncate LOS; the composite PLOS/death sensitivity analysis produced concordant results. Finally, the study cohort overlaps with our previous PEP-focused publication [11]. This overlap is explicitly disclosed; the present study addresses a distinct outcome, uses a different analytical framework, and focuses on hospitalization rather than the incidence or predictors of PEP.

Overall, prolonged hospitalization among patients undergoing ERCP was associated with the combined effects of acute presentation, underlying disease, and patient vulnerability. Emergency admission was the dominant baseline association, while cholangitis/postoperative conditions, liver disease, age, and malignant obstruction contributed across complementary analyses. PEP was also associated with longer hospitalization, although the magnitude of this association should be interpreted cautiously because of uncertainty in historical adjudication and possible circularity with LOS.

## 5. Conclusions

Study-defined prolonged hospitalization in patients undergoing ERCP was associated primarily with the clinical context of admission, underlying disease, and patient characteristics. Emergency admission, cholangitis or postoperative biliary complications, liver disease, and increasing age were associated with the primary PLOS outcome, while malignant obstruction contributed in complementary and sensitivity analyses. PEP was also associated with longer hospitalization, but this association should not be interpreted causally because the historical diagnostic adjudication rule was not retained and some circularity with LOS cannot be excluded. The findings were broadly robust to alternative LOS thresholds, inclusion of in-hospital death, and exclusion of PEP cases. These explanatory results may inform inpatient planning and future hypothesis generation but do not constitute a validated prediction tool.

## Figures and Tables

**Table 1:** Characteristics of the primary descriptive cohort according to prolonged hospital stay status.

Characteristic	Overall (*n* = 788)	LOS < 12 Days (*n* = 612)	PLOS ≥ 12 Days (*n* = 176)	*p*-Value
Age, years, median (IQR)	66 (56–75.2)	65 (54–75)	68 (59–77)	0.011
Female sex	427 (54.2)	336 (54.9)	91 (51.7)	0.453
Emergency admission	487/787 (61.9)	335/611 (54.8)	152 (86.4)	<0.001
Gallbladder stones	256 (32.5)	196 (32.0)	60 (34.1)	0.606
Post-cholecystectomy status	229 (29.1)	191 (31.2)	38 (21.6)	0.013
Previous ERCP	207 (26.3)	177 (28.9)	30 (17.0)	0.002
History of acute pancreatitis	92 (11.7)	79 (12.9)	13 (7.4)	0.044
Liver disease	25 (3.2)	14 (2.3)	11 (6.2)	0.008
Diabetes mellitus	141 (17.9)	102 (16.7)	39 (22.2)	0.094
Obesity	47 (6.0)	35 (5.7)	12 (6.8)	0.587
Sphincterotomy	518 (65.7)	387 (63.2)	131 (74.4)	0.006
Post-ERCP pancreatitis	56 (7.1)	29 (4.7)	27 (15.3)	<0.001
**ERCP indication**				0.001
Choledocholithiasis	369 (46.8)	302 (49.3)	67 (38.1)	
Malignant obstruction	243 (30.8)	171 (27.9)	72 (40.9)	
Benign/reintervention	138 (17.5)	114 (18.6)	24 (13.6)	
Cholangitis/postoperative	38 (4.8)	25 (4.1)	13 (7.4)	
**Biliary stent status**				<0.001
No stent	273 (34.6)	220 (35.9)	53 (30.1)	
Plastic stent	389 (49.4)	285 (46.6)	104 (59.1)	
SEMS	56 (7.1)	38 (6.2)	18 (10.2)	
Stent removal	70 (8.9)	69 (11.3)	1 (0.6)	

Data are *n* (%) unless otherwise indicated. Admission mode was unavailable for one patient; denominators are therefore shown explicitly for that variable. *p*-values compare LOS < 12 days with PLOS ≥ 12 days. ERCP, endoscopic retrograde cholangiopancreatography; IQR, interquartile range; LOS, length of stay; PEP, post-ERCP pancreatitis; PLOS, prolonged hospital stay; SEMS, self-expandable metal stent.

**Table 2:** Modified Poisson regression with HC0 robust standard errors for prolonged hospital stay (LOS ≥ 12 days; complete-case *n* = 787).

Variable	Model A aRR (95% CI)	*p*-Value	Model B aRR (95% CI)	*p*-Value
Age per 10 years	1.12 (1.02–1.22)	0.013	1.13 (1.04–1.23)	0.003
Female sex	1.00 (0.77–1.30)	0.985	1.00 (0.77–1.29)	0.981
Emergency admission	3.01 (2.01–4.51)	<0.001	2.96 (1.98–4.45)	<0.001
Gallbladder stones	1.01 (0.74–1.38)	0.940	0.94 (0.68–1.29)	0.698
Post-cholecystectomy status	0.82 (0.58–1.15)	0.254	0.83 (0.59–1.17)	0.289
Previous ERCP	0.69 (0.46–1.04)	0.080	0.70 (0.46–1.07)	0.101
History of acute pancreatitis	0.76 (0.45–1.27)	0.293	0.68 (0.40–1.14)	0.139
Liver disease	1.85 (1.10–3.12)	0.021	1.84 (1.12–3.04)	0.017
Diabetes mellitus	1.11 (0.81–1.52)	0.522	1.10 (0.81–1.48)	0.547
Obesity	0.99 (0.62–1.59)	0.981	1.02 (0.62–1.66)	0.947
Indication: malignant obstruction	1.29 (0.91–1.82)	0.149	1.33 (0.95–1.88)	0.101
Indication: benign/reintervention	1.32 (0.88–1.98)	0.185	1.36 (0.91–2.02)	0.130
Indication: cholangitis/postoperative	2.71 (1.57–4.66)	<0.001	2.36 (1.29–4.30)	0.005
Sphincterotomy	0.96 (0.69–1.34)	0.829	0.90 (0.65–1.26)	0.545
Stent: plastic	1.10 (0.78–1.53)	0.593	1.06 (0.76–1.48)	0.728
Stent: SEMS	1.21 (0.71–2.06)	0.477	1.22 (0.72–2.08)	0.465
Stent: removal	0.15 (0.02–1.12)	0.064	0.15 (0.02–1.04)	0.055
Recorded PEP	—	—	2.34 (1.67–3.29)	<0.001

**Table 3:** Fully adjusted secondary analyses of total hospital length of stay (complete-case *n* = 787).

Variable	NB IRR	95% CI	*p*-Value	Median Difference, Days	95% CI	*p*-Value
Age per 10 years	1.05	1.02–1.08	<0.001	+0.35	+0.16 to +0.54	<0.001
Female sex	0.97	0.89–1.05	0.417	+0.04	−0.54 to +0.62	0.893
Emergency admission	1.74	1.59–1.90	<0.001	+3.95	+3.34 to +4.57	<0.001
Gallbladder stones	1.03	0.93–1.14	0.581	+0.11	−0.59 to +0.82	0.752
Post-cholecystectomy status	0.99	0.90–1.10	0.898	+0.18	−0.52 to +0.88	0.613
Previous ERCP	0.91	0.82–1.02	0.102	−0.81	−1.55 to −0.06	0.034
History of acute pancreatitis	0.92	0.81–1.05	0.223	+0.01	−0.88 to +0.90	0.978
Liver disease	1.35	1.09–1.68	0.006	+1.22	−0.37 to +2.81	0.133
Diabetes mellitus	1.04	0.94–1.16	0.469	+0.85	+0.11 to +1.59	0.024
Obesity	0.93	0.78–1.11	0.428	−0.30	−1.50 to +0.89	0.617
Indication: malignant obstruction	1.16	1.04–1.30	0.007	+1.06	+0.29 to +1.83	0.007
Indication: benign/ reintervention	1.01	0.89–1.14	0.934	+0.42	−0.41 to +1.24	0.325
Indication: cholangitis/ postoperative	1.45	1.20–1.76	<0.001	+2.47	+1.07 to +3.87	<0.001
Sphincterotomy	1.05	0.95–1.16	0.345	+0.50	−0.20 to +1.19	0.160
Stent: plastic	1.01	0.92–1.11	0.846	+0.00	−0.68 to +0.69	0.991
Stent: SEMS	1.03	0.86–1.23	0.751	+0.74	−0.52 to +2.00	0.251
Stent: removal	0.71	0.59–0.86	<0.001	−0.39	−1.57 to +0.79	0.517
Recorded PEP	1.62	1.40–1.87	<0.001	+3.46	+2.36 to +4.56	<0.001

Negative binomial regression used an NB2 variance structure and log link; results are incidence rate ratios (IRRs). Median quantile regression was performed at τ = 0.50; coefficients are adjusted differences in median LOS in days. Choledocholithiasis and no stent were reference categories. CI, confidence interval; IRR, incidence rate ratio; NB, negative binomial; PEP, post-ERCP pancreatitis; SEMS, self-expandable metal stent.

**Table 4:** Sensitivity analyses for prolonged hospital stay and alternative hospitalization outcomes.

Analysis	N (Events)	Age/10 y	Emergency Admission	Liver Disease	Malignant Obstruction	Cholangitis/Postoperative	Recorded PEP
Primary PLOS ≥ 12 d, Model B	787 (176)	1.13 (1.04–1.23)	2.96 (1.98–4.45)	1.84 (1.12–3.04)	1.33 (0.95–1.88)	2.36 (1.29–4.30)	2.34 (1.67–3.29)
LOS > 7 d, Model B	787 (364)	1.11 (1.06–1.17)	2.41 (1.91–3.04)	1.12 (0.78–1.61)	1.28 (1.08–1.53)	1.90 (1.35–2.69)	1.61 (1.31–1.97)
LOS > 14 d, Model B	787 (105)	1.13 (1.00–1.27)	2.57 (1.55–4.27)	2.10 (1.02–4.32)	1.23 (0.75–2.01)	2.37 (1.09–5.16)	2.79 (1.71–4.56)
PEP excluded, Model A	731 (149)	1.20 (1.10–1.32)	3.22 (2.05–5.07)	1.53 (0.85–2.75)	1.50 (1.02–2.22)	3.83 (2.20–6.69)	—
PLOS ≥ 12 d or death, Model B	804 (193)	1.13 (1.05–1.22)	2.56 (1.79–3.65)	1.84 (1.17–2.89)	1.59 (1.14–2.21)	2.30 (1.32–3.99)	2.34 (1.71–3.20)

Values are adjusted risk ratios (95% CIs). All sensitivity models included the full covariate set used in the corresponding primary model; only selected clinically relevant coefficients are displayed for readability. The PEP-excluded analysis refitted Model A after excluding all patients with PEP. Age effects are per 10-year increase. PEP, post-ERCP pancreatitis; PLOS, prolonged hospital stay.

**Table 5:** Descriptive model-adequacy diagnostics.

Model	*n*	AIC	Pseudo-R^2^	Additional Adequacy Measures
Modified Poisson, Model A	787	831.7	0.095	Deviance explained 15.8%; RMSE 0.393
Modified Poisson, Model B	787	821.3	0.109	Deviance explained 18.2%; RMSE 0.389
Negative binomial NB2, Model B	787	4468.4	0.066	RMSE 5.50 days; Pearson χ^2^/df 1.24; alpha 0.189
Median quantile regression, Model B	787	—	0.190	Koenker–Machado pseudo-R^2^ at τ = 0.50

Pseudo-R^2^ denotes McFadden pseudo-R^2^ for modified Poisson and negative binomial models and Koenker–Machado pseudo-R^2^ for median quantile regression. Pseudo-R^2^ values are model-family specific and should not be compared directly across the different model types. RMSE values describe in-sample fit and were not used as evidence of predictive validation. AIC, Akaike information criterion; RMSE, root mean square error.

## Data Availability

De-identified data supporting the findings of this study are available from the corresponding author upon reasonable request, subject to applicable institutional and ethical requirements.
